# Multi-grade fuzzy assessment framework for software professionals in work-from-home mode during and post-COVID-19 era

**DOI:** 10.1186/s43093-021-00057-w

**Published:** 2021-03-15

**Authors:** M. Suresh, Kavya Gopakumar

**Affiliations:** Amrita School of Business, (AACSB Accredited Business School), Amrita Vishwa Vidyapeetham (University), Coimbatore, 641 112 India

**Keywords:** Work-from-home, Online work during COVID-19, Software professionals, Work effectiveness, Multi-grade fuzzy, Importance–performance analysis

## Abstract

The pandemic novel Coronavirus disease and the resulting lockdowns have contributed to major economic disturbances around the world, forcing organisations to extend the work-from-home (WFH) option to their employees wherever feasible. The current major challenge of this option is maintaining the efficiency and productivity of the employees across the organisations. It is therefore important to understand the impact of this make-shift arrangement of WFH policy and their underlying effects that may affect the efficiency of employees and hence their output levels. This is a distinctive approach to develop a unique framework for efficiency index computation by evaluating the efficiency levels of WFH mode in software organisations using multi-grade fuzzy approach and importance–performance analysis. In turn, this would help to determine the crucial attributes that require improvement to increase the efficiency levels of employees concerned. In this study, a case project has been assessed and it was observed that the efficiency index of WFH accounts to 4.92, which is in between the range of (4.01–6) specified as ‘Efficient’. The framework can be used on a periodic basis to help software organisations to continuously improve their WFH efficiency level.

## Introduction

The unprecedented COVID-19 pandemic has disrupted the business ecosystem in many ways. Many organisations across the globe have been left with a choice to have flexible working arrangements like working from home (WFH), especially the Information Technology (IT)-based organisations. During lockdown, the IT industry has shifted seamlessly to the work-from-home model, offering business continuity to customers without reducing efficiency or productivity. Several business leaders find WFH to be a permanent feature, and have started to analyse its advantages and drawbacks. Recent developments in the information and communication technologies have made it easier to perform tasks outside the work-environment. Good internet connectivity and user-friendly machines enable software professionals in the IT industry to work from home. But how this work-model transition has impacted the employees and the respective stakeholders is a concern to be studied upon. It is important to analyse the efficiency and performance of software professionals in the IT industry in order to evaluate the efficiency of WFH model. Only when efficiency improves, the performance improves. This results in an increase in productivity levels and performance, as these factors are interconnected and are essential to achieve the goals of a company.

This research is conducted to study the efficiency of software professionals who are working from home due to this COVID-19 pandemic. The attributes related to efficiency have been drawn from review of various literatures and also by taking note of expert opinions. The attributes were then rated according to its effect on efficiency, in order to find the weakest among them. An MGF assessment model is necessary to examine how the WFH model has affected a team’s or project’s efficiency and productivity. Through this study, the weaker attributes which are required to be improved in order to increase the efficiency will be assessed and proper suggestions will be provided to modify the same. Thus, a distinctive framework was developed which can be adopted by any organisation in the software industry, to evaluate the work-from-home efficiency levels of a particular team or a project.

## Literature review

A key policy implemented during the COVID-19 pandemic was 'social distancing' [19] in the absence of a vaccine or widespread testing which allows employees in many jobs to work from home wherever feasible. In addition to this, returning to work is likely to occur more slowly for those jobs which involves a large degree of personal closeness to others [22]. According to Felstead et al. [9], the option of working at home is more likely to be available in the public sector, in large establishments and also in those work environments where individuals are responsible for the quality of their own production.

As per Lakshmi et al. [18], work-from-home is one such activity that human resource managers should pursue to recruit and retain high-quality employees with benefits that go beyond the simple work–life balance. The organisations must consider the relevant perspectives and steps that will help both the organisations and their employees to plan and provide maximum value by providing an option to work from home. WFH provides temporary flexibility and flexibility to the worker when choosing working conditions [11].

Where occupational characteristics outweigh individual characteristics, shifting to work-from-home can depend on the average level of productivity at home versus at work, and does not require much selection, training and monitoring if workers choose to work from home [17]. From the perspective of work-from-home, most of the variable organisational and task characteristics were correlated with the outcome measures including productivity, while the individual and household variables were less closely related [3].

According to the study conducted by Duxbury et al. [8], computer-supported supplemental work-at-home (SWAH) provides advantages for organisations to promote the adoption of home technology for their employees. The organisations that provide computer equipment for their employees to work from home will be benefiting more in terms of employees’ productivity and profit. If a company is not providing proper technical tools that fits the job that needs to be done in a virtual office, it can adversely affect the productivity of the employees [15]. There should be a well-designed training program for the employees and also for the leaders which includes detailed instructions on the usage of technology and also on the social and psychological changes to be made by the respective organisations in this regard.

The study conducted by Venkatesh and Vitalari [26] says that autonomy, flexibility and improved efficiency are the key reasons behind working from home. Another major defining factor is the portability. Hence, while designing jobs, the organisation must consider these factors to make the work-from-home facility more effective. The employers should be more conscious of the benefits that might be obtained from a sound and solid family lives of their employees, without dismissing these issues, if it were solely a personal problem of an individual employee alone [13].

Programmers consider telecommuting as an alternative to improve job satisfaction as they have a feeling that it would result in ‘improved morale’. In a study conducted by DeSanctis [6], it was advised to carry out more research on how organisations can select the most appropriate alternative among the following options such as flexitime, working in a satellite facility and telecommuting so that it is favourable to enhance the productivity of their programmers.

The economic and psychological benefits gained from a family-friendly workplace include recruiting and retaining skilled-staff, high levels of physical and psychological well-being of employees, lower absenteeism, lesser sick-leave rates and increased productivity [4].

There are many theories related to how employees manage themselves based on the circumstances that they have to undergo, in order to achieve the targeted performance in their work. Also, in the face of high job demands, employees either adopt performance protection strategies (e.g. mobilisation of additional mental effort related with additional costs) or accept a decrease in insidious performance (with no cost increase). The former is termed active coping mode, and the latter the passive coping mode [5, 16].

According to the study conducted by Olson [23], the count of management and professional workers making use of information technology to stay in touch twenty-four hours a day, to extend the work day and the workplace, and to provide rapid responses is increasing day-by-day. This led to the usage of information technology to support remote collaboration, particularly in those companies which lack a traditional bureaucratic hierarchy structure.

The MGF assessment of efficiency of WFH model among software professionals is inevitably required at various stages of projects in software organisations. Adopting WFH strategy for all software professionals is a new research area, and since only a very little exploration has happened in this field, this has led to a motivation for our study. The current study attempts to apply multi-grade fuzzy to assess the efficiency of software professionals to achieve improvement in overall performance of software projects, during and post-COVID-19 era.

In this paper, WFH enablers, criteria and their attributes were identified and a conceptual framework was developed for the assessment of the efficiency level of WFH in COVID-19 era by using a multi-grade fuzzy approach. The major objectives are shown below:

- To identify the WFH enablers, criteria and attributes of various software professionals.
- To develop a multi-grade fuzzy based assessment framework for measuring the efficiency of WFH of software professionals.
- To identify the weaker attributes of WFH of software professionals in the case of a software project organisation and to propose suggestions for improvement.

The aforementioned objectives were converted into the following research questions (RQ):

- RQ1: How to measure the efficiency of WFH of software professionals?
- RQ2: What are the attributes which affect efficiency of WFH mode in software projects?
- RQ3: How to improve the weaker attributes to enhance the efficiency of WFH model?

In order to answer the questions listed above, an assessment study was conducted. The measure of efficiency so obtained would assist the project managers to achieve the scope of their project. Additionally, a periodical assessment on efficiency of WFH level of software professionals would notably enhance their continuous improvement in performance and services.

The rest of the paper is organised as follows. "Methods" section includes research methodology with a multi-grade fuzzy approach and an analysis on IPA. "Results and discussions" section is about the results and discussion with suggestions to improve weaker attributes of the case project. "Practical/Managerial implications" enlists the practical implications, while "Conclusion" section deals with the conclusion.

## Methods

### Case organisation

The case software organisation is located in India, and it encourages its share of employees to work from home, due to the dreaded COVID-19 situation. This particular organisation runs multiple projects parallelly. An assessment study was conducted on one among these case projects, where all members of the team were working from their home, from various locations across India and overseas. The objective of this framework was to assess the overall efficiency of their work through WFH mode in the respective case project.

### Multi-grade fuzzy

The multi-grade fuzzy approach is extensively applied in manufacturing and service sectors for assessment of leanness, agility, marketing flexibility, safety practice level and service level [1, 12, 21, 24, 27–32]. The current study utilises multi-grade fuzzy to assess the efficiency of the work-from-home model among software professionals. The study begins with a review of literature on work-from-home policy and other related attributes across various domains. The new conceptual model was framed on the basis of COVID-19 situation to assess the efficiency levels with 3 enablers, 11 criteria and 27 attributes (Table 1). The enablers, criteria and attributes were finalised based on an interview with the experts’ panel. A qualitative data collection was performed to amass weightage from a panel of five experts from various software organisations and ratings were procured from yet another set comprising five team members, working under the case project in software organisation across India. The respondents’ profile is shown in Table 2. The interview lasted for 30–45 min, and a brief introduction was given to the respondents on enablers, criteria and attributes. The experts’ opinion was captured in linguistic variables and was converted into an equivalent fuzzy scale using Table 3. While allocating weights, reverse ranking was performed for negative attributes, as their effect might tend to decrease the efficiency of software professionals (Table 3).

**Table 1:** Conceptual model for work-from-home for software professionals

Enabler	Criteria	Attributes
Human perspective (O1)	Work–life balance (O11)	Motivation (O111)
		Stress (O112)
		Self-efficacy (O113)
		Readiness to prioritise both work and life (O114)
	Health issues (O12)	Ergonomic issues (O121)
		Long screen time (O122)
	Time management (O13)	Working hours (O131)
		Personal time (O132)
		Time lost in transportation (O133)
Technical perspective (O2)	Technical knowledge (O21)	Software knowledge (O211)
		Setting up of VPN and other network-related needs (O212)
	Training and development (O22)	Proper training and development (O221)
		Knowledge gap (O222)
	Network issues (O23)	Unavailability of network at home (O231)
		Network speed fluctuations (O232)
	Hardware issues (O24)	Unavailability of required hardware (O241)
		Incompatible hardware (O242)
Management perspective (O3)	Supervisory support (O31)	’Over the shoulder’ supervision (O311)
		Supervisor’s/Manager’s availability (O312)
	Colleague support (O32)	Mismatch of intermission period among colleagues (O321)
		Spending quality time discussing personal and professional life (O322)
		Peer-to-peer relationship (O323)
	Rewards and recognition (O33)	Opportunities for personal/professional development (O331)
		Appreciation and other perks (O332)
	Team coordination (O34)	Miscommunication among the team members (O341)
		Team-building activities (O342)
		Feeling of connectedness to the team and organisation (O343)

**Table 2:** Respondents profile

Respondent No	Designation	No. of years’ work experience	Location	Company
For enabler, criteria and attributes weightage				
RS-1	IT analyst	6	Hyderabad, India	Multinational company
RS-2	Software engineer	3	Bangalore, India	Multinational company
RS-3	Cyber security engineer	5	Kochi, India	Multinational company
RS-4	Developer	5	Kolkata, India	Multinational company
RS-5	Senior engineer	8	Bangalore, India	Multinational company
For attributes rating				
E-1	Developer	3	Chennai, India	Multinational company
E-2	Developer	5	Chennai, India	Multinational company
E-3	Business analyst	8	Chennai, India	Multinational company
E-4	Developer	4	Chennai, India	Multinational company
E-5	Team lead	10	Chennai, India	Multinational company

**Table 3:** Rating and weight-scale for work-from-home for software professionals

Sl.no	Attributes rating				Enabler, Criteria and attributes weightage	
	For positive attributes		For negative attributes			
	Linguistic variable (*O*_*ijk*_)	Rating (Fuzzy scale)	Linguistic variable (*O*_*ijk*_)	Rating	Linguistic variable	Weightage (*W*_*ijk*_/*W*_*ij*_/*W*_*i*_) (Fuzzy scale)
1	Worst	1	Outstanding	1	No importance	1
2	Very very poor	2	Very very good	2	Very less important	2
3	Very poor	3	Very good	3	Less important	3
4	Poor	4	Good	4	Moderate important	4
5	Fair	5	Highly fair	5	Important	5
6	Highly fair	6	Fair	6	Highly important	6
7	Good	7	Poor	7	Very high important	7
8	Very good	8	Very poor	8	Very very high important	8
9	Very very good	9	Very very poor	9	Extremely important	9
10	Outstanding	10	Worst	10	Very extremely important	10

Here, the efficiency assessment index of software projects is represented as *I*. It is the product of overall assessment level of ratings based on each driver (*R*) and the overall weights (*W*) given by the experts. The equation for efficiency index is

$$ I = W \times R $$

The assessment scale has been graded into five levels since every factor involves fuzzy determination. *I* = (10, 8, 6, 4, 2). 8–10 represents ‘Extremely Efficient’, 6–8 represents ‘Highly Efficient’, 4–6 represents ‘Efficient’, 2–4 represents ‘Moderately Efficient’ and less than 2 denotes ‘Quite Inefficient’. Table 4 lists the assessment model weights and performance rating from the experts.

**Table 4:** Normalised weights and experts’ rating of the case project

*O*_*i*_	*O*_*ij*_	*O*_*ijk*_	*E*1	*E*2	*E*3	*E*4	*E*5	*W*_*ij*_	*W*_*i*_	*W*
O1	O11	O111	9	5	4	8	3	0.238462	0.343511	0.380952
		O112	5	8	1	3	2	0.261538		
		O113	7	7	4	8	9	0.215385		
		O114	4	3	2	8	3	0.284615		
	O12	O121	4	3	2	2	1	0.490566	0.343511	
		O122	5	4	2	2	2	0.509434		
	O13	O131	3	4	4	3	7	0.28	0.312977	
		O132	4	5	8	4	7	0.34		
		O133	7	9	9	9	5	0.38		
O2	O21	O211	6	7	8	8	6	0.544118	0.25	0.309524
		O212	6	5	4	2	6	0.455882		
	O22	O221	7	5	6	8	9	0.575758	0.261111	
		O222	6	6	5	3	3	0.424242		
	O23	O231	3	4	4	6	4	0.512195	0.261111	
		O232	3	6	6	2	6	0.487805		
	O24	O241	3	1	2	1	8	0.514706	0.227778	
		O242	3	1	4	7	9	0.485294		
O3	O31	O311	5	4	4	8	8	0.512195	0.235294	0.309524
		O312	7	10	8	9	9	0.487805		
	O32	O321	2	5	4	6	3	0.424658	0.247059	
		O322	3	2	4	1	1	0.287671		
		O323	5	2	4	3	6	0.287671		
	O33	O331	4	7	9	7	8	0.581818	0.241176	
		O332	7	3	4	2	4	0.418182		
	O34	O341	5	7	4	3	2	0.233766	0.276471	
		O342	4	1	2	1	1	0.324675		
		O343	7	4	8	9	8	0.441558		

#### First-level calculation

The first-level calculation done for ‘Work–life balance (O11)’ criterion is given below. Weights concerning to ‘Work–life balance’ criterion is *W*_11_ = [0.238, 0.261, 0.215, 0.284]

$$ R_{11} = \left[ {\begin{array}{*{20}c} 9 \quad & 5  \quad & 4  \quad & 8  \quad & 3 \\ 5  \quad & 8  \quad & 1  \quad & 3  \quad & 2 \\ 7  \quad & 7  \quad & 4  \quad & 8  \quad & 9 \\ 4 \quad  & \quad  3 \quad  & 2  \quad & 8  \quad & 3 \\ \end{array} } \right] $$

‘Work–life balance’ calculation Index, *I*_11_ = *W*_11_ × *R*_11_

$$ I_{11} = \left[ {6.1, \, 5.65, \, 2.65, \, 6.69, \, 4.03} \right] $$

Using similar principle, remaining criterion indices were obtained as follows:

$$ \begin{aligned} I_{12} & = \, \left[ {4.51, \, 3.51, \, 2, \, 2, \, 1.51} \right] \\ I_{13} & = \, \left[ {4.86, \, 6.24, \, 7.26, \, 5.62, \, 6.24} \right] \\ I_{21} & = \, \left[ {6, \, 6.08, \, 6.18, \, 5.26, \, 6} \right] \\ I_{22} & = \, \left[ {6.57, \, 5.42, \, 5.57, \, 5.87, \, 6.45} \right] \\ I_{23} & = \, \left[ {3, \, 4.97, \, 4.97, \, 4.04, \, 4.97} \right] \\ I_{24} & = \, \left[ {3, \, 1, \, 2.97, \, 3.91, \, 8.48} \right] \\ I_{31} & = \, \left[ {5.97, \, 6.92, \, 5.95, \, 8.48, \, 8.48} \right] \\ I_{32} & = \, \left[ {3.15, \, 3.27, \, 4, \, 3.69, \, 3.29} \right] \\ I_{33} & = \, \left[ {5.25, \, 5.32, \, 6.9, \, 4.9, \, 6.33} \right] \\ I_{34} & = \, \left[ {5.55, \, 3.72, \, 5.11, \, 5, \, 4.32} \right] \\ \end{aligned} $$

#### Second-level calculation

The second-level calculation done for ‘Human perspective (O1)’ enabler is given below. Weights concerning to ‘Human perspective’ enabler is *W*_1_ = [0.343, 0.343, 0.312]

$$ R_{1} = \left[ {\begin{array}{*{20}c} {6.1} &\quad {5.65} &\quad {2.65} &\quad {6.69} &\quad {4.03} \\ {4.51} &\quad {3.51} &\quad 2 &\quad 2 &\quad {1.51} \\ {4.86} &\quad {6.24} &\quad {7.26} &\quad {5.62} &\quad {6.24} \\ \end{array} } \right] $$

‘Human Perspective’ calculation Index, *I*_1_ = *W*_1_ × *R*_1_

$$ I_{1} = \, \left[ {5.16, \, 5.10, \, 3.87, \, 4.74, \, 3.86} \right] $$

Using similar principle, remaining enabler indices were obtained as follows:

$$ \begin{gathered} I_{2} = \, \left[ {4.68, \, 4.46, \, 4.97, \, 4.8, \, 6.41} \right] \hfill \\ I_{3} = \, \left[ {4.99, \, 4.75, \, 5.47, \, 5.48, \, 5.53} \right] \hfill \\ \end{gathered} $$

#### Third-level calculation

The third-level calculation done for the ‘Efficiency index’ of WFH is given below. Weights concerning the overall ‘efficiency’ are *W* = [0.381, 0.309, 0.309]

$$ R_{1} = \left[ {\begin{array}{*{20}c} {5.16} &\quad {5.10} &\quad {3.87} &\quad {4.74} &\quad {3.86} \\ {4.68} &\quad {4.46} &\quad {4.97} &\quad {4.8} &\quad {6.41} \\ {4.99} &\quad {4.75} &\quad {5.47} &\quad {5.48} &\quad {5.53} \\ \end{array} } \right] $$

Overall efficiency calculation Index, *I* = *W* × *R*

$$ \begin{aligned} I & = \left[ {4.96, \, 4.79, \, 4.70, \, 4.99, \, 5.17} \right] \\ I & = \left( {4.96 \, + \, 4.79 \, + \, 4.70 \, + \, 4.99 \, + \, 5.17} \right)/5 \to 4.92 \\ I & = 4.92 \in \left( {4, \, 6} \right) \to {\text{'Efficient'}} \\ \end{aligned} $$

For the respective case project under study, the efficiency index accounts to 4.92, indicative of the fact that the project employees are ‘efficient’. Up next, the IPA analysis was carried out for classifying the attributes based on their importance and performance.

### Importance–performance analysis (IPA)

**Fig. 1:**
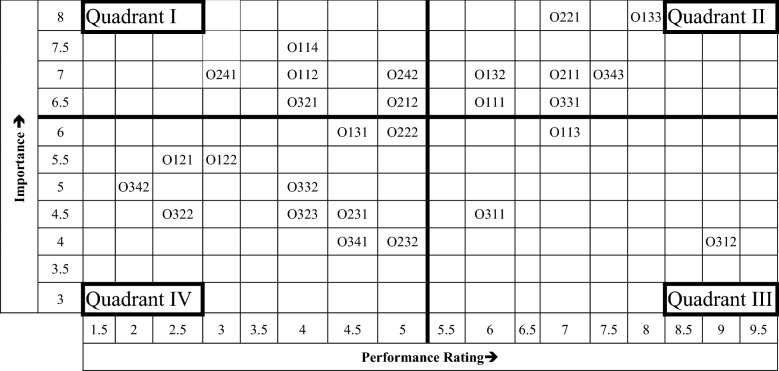
IPA for efficiency assessment attributes of case project

Importance–performance analysis (IPA) is widely used to classify attributes based on weaker attributes (attention required), good-performance attributes (keep it up), over-performance attributes (re-allocation of resources to reduce performance) and low-priority attributes (only less attention required), etc. It is extensively applied in manufacturing and service sectors to classify various attributes or to identify their priority [2, 7, 10, 14, 20, 25]. In IPA, the horizontal axis marks the performance of attributes and the vertical axis denotes their importance. In this case project, IPA of mean of *x*-axis is 4.89 and the mean of *y*-axis is 5.72 as per the perpendicular line given in Fig. 1.

Quadrant I (Weaker attributes): The attributes in this quadrant need immediate attention by the case project manager in order to improve the employees’ efficiency. The attributes are stress, readiness to prioritise work and life, setting up of VPN and other network-related needs, unavailability of required hardware or incompatible hardware, and mismatch of intermission period among colleagues.

Quadrant II (Keep up the good work): The attributes in this quadrant are indeed necessary to keep up the efficacy of employees. They are motivation, more personal time, time lost in transportation, software knowledge, proper training and development, opportunities for personal/professional development, feeling of connectedness to the team and organisation.

Quadrant III (Possible overkill): The importance of these attributes is quite low since the performance rating is over the top. Those attributes include self-efficacy, ‘Over the shoulder’ supervision, supervisor’s/manager’s availability.

Quadrant IV (Low priority): The attributes in this quadrant are of low importance, and requires only lesser attention. It includes ergonomic issues, long screen time, long working hours, knowledge gap, unavailability of network at homes, network speed fluctuations, spending quality time discussing personal and professional life, peer-to-peer relationship, appreciation and other perks, miscommunication among the team, and team-building activities.

## Results and discussion

In an effort to measure the efficiency of employees working from home, the efficiency index was calculated as 4.92, which belongs to the range of (4.01–6) specified as ‘Efficient’. The existing efficiency level requires further improvement on weaker attributes in order to achieve the level earmarked as ‘Extremely Efficient’. This in turn, requires a detailed management of actions to be undertaken to improve the weaker attributes and thereby certain measures to enhance the efficiency of employees. According to the current study, the employees’ feels motivated, enjoys their personal time and are equipped with proper knowledge about the software that they are using. They were getting the advantage of time that they tend to lose early, during their transport between home and the office. Provisions for proper training and development not only helps them to improve and enhance their efficiency but also provides good opportunities for their personal and professional development. Though miles apart, the members would still have the feeling of connectedness to their team and organisation, keeping them motivated to work cooperatively to achieve targets of the company.

The weaker attributes demand proper attention and strategies to improve them. Measures taken to improve these attributes not only helps to increase the efficiency of individual employees but also of the team as a whole. Engaging in stress-relieving activities, prioritising both work and life, proper rectification of network/software/hardware-related issues and coordination among team members demands major attention in accordance with IPA analysis performed as a part of this research. Suggestions to improve the weaker attributes are listed in Table 5.

**Table 5:** Identified weaker attributes and decisions suggested for their improvement

Weaker attributes	Suggestions for improvement
Stress	Find a right place at home to work Take a break in between the working hours Relaxing in between the working time by listening to songs, doing yoga, ergonomic exercises, etc. Organise the way of working Get support from friends and family Learn new hobbies
Readiness to prioritise both work and life	Fix proper boundaries while devoting time exclusively for work and personal life To-do list prioritising tasks to be done Proper log-in and log-off time (fixed working hours) Learn to say ‘No’ to those tasks which you are not ready to take up Avoid non-value-added activities during the working hours Support from the co-workers
Setting up of VPN and other network-related needs	The company has to provide proper network connection devices/reimburse if personal devices are being used Migrate to a better network service provider Proper power-backup at home An alternative should be available in case of unpredicted network issues hampering the work
Unavailability of required hardware	Office/Client laptop is preferred over personal laptops The company should plan for digital transformation reducing the need for physical hardware like printers, scanners, etc.
Incompatible hardware	Patching/software updates should be planned as per the availability The support team should be available as and when it is required to provide a solution If needed, monitors should be provided so that the employees can avoid stress on eyes by looking into small laptop screens
Mismatch of intermission period among colleagues	The daily activities/tasks should be planned and communicated among the team members well in advance Maintenance of proper timetable The team has to work on the same shift whenever possible The team members have to be connected to better network in order to avoid non-value-added activities due to network issues

## Practical/Managerial implications

The present study initially identifies the enablers, criteria and attributes which assists to measure the efficiency and productivity of employees from various software/Information Technology domains of an organisation, working from home during the COVID-19 period. Responses from various employees including a panel of experts from varied projects were recorded based on the attributes and were taken thereafter for a detailed IPA analysis. The output of IPA analysis identifies the weaker attributes as stress, readiness to prioritise both work and life, setting up of VPN and other network-related needs, unavailability of required hardware or incompatible hardware and mismatch of intermission period among colleagues, etc.

It is necessary for an organisation to take note of these weaker attributes in order to improve the efficiency of employees within a team and across teams to guarantee the achievement of maximum productivity. The suggestions for improvement should be taken into consideration to assist an organisation to achieve its goals through an efficient work-from-home policy for its employees during the COVID-19 situation. Both the team members and the panel of experts should be well-aware of these attributes which would encourage them to work more efficiently and effectively. The managers and the organisations must ensure that the stronger attributes and their related criteria are continuing as the way it is at present. Hence, with cooperation from the team as well as the organisation, one can succeed the challenges that hinder efficiency in a work-from-home model.

## Conclusion

The unprecedented COVID-19 situation has forced many organisations to practise work-from-home policy for their employees wherever feasible. Through IPA analysis, the study identifies certain weaker attributes that demand immediate improvement. The enablers, criteria and attributes identified were manipulated and were used for IPA analysis. In order to evaluate the current efficiency level of employees working in an Information Technology (software) domain, this study can be considered as a base for further research on remaining domains as well. The suggested new approach would definitely improve the efficiency of employees within a team as well as across teams and would also help in attaining both personal and organisational goals. Management should be ready to accommodate these changes which would further help to improve the efficiency and in turn, the productivity of the employees both at team and organisational level.

## Data Availability

The datasets used and/or analysed during the current study are presented in Table 3 of this paper.

## Abbreviations

*O*_*ijk*_: Attributes rating
*W*_*ijk*_: Attributes weightage
*W*_*ij*_: Criteria weightage
*W*_*i*_: Enabler weightage
